# Review of a Study on Late Referral to a Palliative Care Consultation Service: Length of Stay and In-Hospital Mortality Outcomes

**Published:** 2015-11-01

**Authors:** Regina M. Fink

**Affiliations:** University of Colorado, Aurora, Colorado

According to the National Consensus Project, palliative care is defined as "patient/family-centered care that optimizes quality of life by anticipating, preventing, minimizing, and treating suffering. Palliative care, offered throughout the continuum of illness, involves addressing physical, intellectual, emotional, social, and spiritual needs and to facilitate patient autonomy, access to information, and choice" (National Consensus Project, 2013). Palliative care is typically offered as an interdisciplinary team approach including physicians, nurses, social workers, chaplains, and specialists in other disciplines to manage pain and symptoms. The Institute of Medicine report "Dying in America" on the quality of care in the United States and its implications for the field of palliative care suggests there is a need to provide palliative care to oncology patients, specifically introducing the concept earlier in the disease trajectory (Institute of Medicine, 2014; Scarborough & Morrison, 2014).

Unfortunately, palliative care is often only offered late in the course of disease after curative measures have been exhausted. This concern of late referral to palliative care consultation is one that plagues many inpatient and outpatient palliative care consultative teams, often leading to suboptimal pain and symptom management, increased suffering, failure to discuss or adhere to advance care planning, and unplanned hospital deaths (Fischer et al., 2006; Teno et al., 2013). However, rather than encouraging a more integrated approach combining palliative care with life-prolonging therapy for patients with serious, advanced illness, current medical care often follows a separate pattern of cure-focused care followed by palliative care only when cure is no longer possible.

The American Society of Clinical Oncology and the Oncology Nursing Society have published position statements on palliative care, suggesting that palliative care can benefit all oncology patients and should be considered early in the course of illness for any individual with metastatic cancer and/or a high symptom burden (Smith et al., 2012; Oncology Nursing Society, 2014).

## THE STUDY: A SYNOPSIS

In a well-written article in the *Journal of Community and Supportive Oncology*, Humphreys and Harman provided a current and extensive literature review stating the need for early palliative care, examining the barriers and challenges to receiving timely inpatient palliative care referrals, and calling attention to the need for understanding health-care outcome differences in oncology patients who receive early vs. late palliative care referral (Humphreys & Harman, 2014). The goal of their retrospective cohort study was to determine whether an inconsistency occurs in health outcomes (hospital length of stay, morbidity, and mortality) for 1,225 oncology Stanford Hospital inpatients who were referred "early vs. late" to the palliative care inpatient consultation service. The time to referral was operationalized as the time calculated from the day of hospital admission to the day of inpatient palliative care referral. Early referral patients were those referred within 1 week of hospital admission; late referral patients were those referred later than 1 week. A secondary study goal was to determine whether policy development could be used to encourage health-care providers to refer to palliative care sooner.

This study’s major strength is the large sample size and the extensive ongoing database, which was developed and maintained by the palliative care inpatient service for approximately 4 years. Data were analyzed using descriptive statistics, tests of difference and association, and regression. The majority of individuals referred to the palliative care service had solid tumor metastatic disease (84%), followed by individuals post bone marrow transplantation (9%) and those with hematologic malignancies (7%).

Some of both groups’ (early vs. late referral) demographics were similar (number of do-not-resuscitate orders, number of recent inpatient admissions, and gender). However, individuals who were referred late to palliative care tended to be younger, were more likely to be white and less likely to be Asian, more often were from an intensive care setting, and were more likely to die in the hospital.

The mean time for referral to palliative care was 2.5 days in the early referral group vs. 21.4 days in the late referral group. In addition, those individuals who were referred early had significantly shorter mean lengths of stay (4.5 days) and lower in-hospital mortality compared with patients’ length of stay (7.4 days), and those who were referred late had higher in-hospital mortality. Waiting at least 1 week to refer to palliative care was associated with the increased odds of a patient’s dying in the hospital vs. being discharged alive by a factor of 3.04 (*p* = .001).

## STUDY LIMITATIONS

A few study limitations that may have influenced this study’s results must be considered. Unfortunately, the specific cancer diagnosis and stage were not delineated in the database. Cancer type and disease status (stage or grade of tumor) may have influenced health outcomes, namely, survivor or decedent status. It is well known that some cancers are more aggressive than others, may not have been as responsive to therapy, and may have influenced referral time to palliative care. This lack of knowledge of diagnosis may have driven the association with survivorship. For example, those patients status post bone marrow transplant or with a hematologic malignancy may have been in the late referral group, because at the time of admission they may have been thought to be in a potentially treatable situation.

In addition, health insurance status was not documented or analyzed. It is possible that those individuals with better insurance coverage may have been treated more aggressively for their cancer, thus affecting the time to palliative care referral. This study only examined the influence of inpatient palliative care consultation services; the effect of an outpatient program had not been established. Outpatient palliative care actually may decrease inpatient admissions and improve patients’ quality of life.

Lastly, a few other variables may have influenced referral time. They include health-care provider (some physicians are more likely to refer to palliative care than others), unit culture about palliative care (the unit or care area housing the patient including the nurses who worked on the unit), patient/family openness to or knowledge about the palliative care concept and referral, and occurrence of advance care planning discussions. All of these factors are unknown. The number of recent inpatient admissions was a proxy for patient illness burden. Other criteria might have been more appropriate to use to determine illness, morbidity, and prognosis.

The work by Humphreys and Harman suggested that late referral to palliative care may negatively impact health outcomes. It may be that the early referrals were individuals who were known to health-care providers as obviously needing palliative care and had maximum reasonable cancer treatment. In addition, were the later-referral patients those who were believed to be still able to respond to cancer treatment? Regardless, the authors believe that the development and implementation of a hospital policy that boosts early referral to palliative care for patients with advanced cancer might be appropriate to decrease their length of hospital stay, improve quality of life, and provide them and their families with improved satisfaction, as their wishes to be discharged to home sooner will be realized.

## REVIEWER’S THOUGHTS ABOUT CHANGING PRACTICE

Developing an evidence-based policy, algorithm, criteria, or checklist that can enable health-care providers to identify the appropriateness of an oncology patient for referral to both inpatient and outpatient palliative care could be advantageous. Von Gunten (2014) posed a question in an editorial in the Journal of Palliative Medicine: "What if the referral to palliative care was part of the order sets for advanced cancer as a matter of course?"

Involving key stakeholders in the development of a policy, algorithm, checklist, or standard order set is crucial. Conducting a current, evidence-based search for instruments and criteria is the first step. Evaluating what other institutions are currently doing as a standard of practice can also be enlightening. An interdisciplinary team, inclusive of oncologists (medical, surgical, radiation), physician assistants, advanced practice nurses [nurse practitioners, research scientists, and clinical nurse specialists] as well as ambulatory and bedside nurses, hospitalists, social workers, psychologists, and chaplains, may assist in the critique and synthesis of the evidence as a first step in developing or adapting a tool for use in one’s practice setting.

**Helpful Tools**

Various tools already exist. For example, in 2010, the Center to Advance Palliative Care (CAPC) assembled a consensus panel to answer the question, "What criteria should be used for hospital staff to conduct prospective case finding, via a checklist, for individuals with unmet palliative care needs?" As a first important step to identify potential individuals in this gap, and with the aim of creating a systems-based change, the panel reviewed existing palliative care consultation triggers from the literature and current consensus panel member practices (Weissman & Meier, 2011). Two checklists were developed: the first for screening at the time of admission, to identify persons whose conditions clearly warrant a basic palliative care assessment (e.g., life-threatening illness, chronic comorbidities that are serious, failure to thrive) and the second for screening at daily rounds (evolving patient issues, symptom burden worsens, ethical consultation is needed due to a lack of congruence in care planning; Weissman & Meier, 2011).

Research suggests that when physicians are asked to provide a prognosis to individuals, they are overly optimistic by a factor of 3 when predicting survival (Glare et al., 2003). Just as hospitals provide algorithms, scoring instruments, and guidelines to determine which persons are at risk for pressure ulcers, delirium, and catheter-associated urinary tract infections, similar aids may assist clinicians in determining patients in need of palliative care.

One such tool, the CARING criteria, was developed by Fischer and colleagues (2006). The CARING criteria are used to identify seriously ill patients who have a high likelihood of death within 1 year and may benefit most from incorporating palliative care and end-of-life discussions into the care plan. A retrospective medical record review validated a set of five prognostic criteria in 895 Veterans Administration Medical Center patients at the time of hospital admission.

The key criteria are as follows: C (primary cancer diagnosis), A (at least two admissions to the hospital for a chronic illness in the past year), R (nursing home resident), I (intensive care unit admission with multiorgan failure), NG (noncancer hospice [meeting at least two of the National Hospice and Palliative Care Organization’s Guidelines]). This simple mnemonic may be useful to help clinicians easily identify patients with a limited life expectancy who may benefit from an early palliative care referral. It can also be used as a measure to determine outcomes when conducting research and quality-improvement projects and evaluating individual practice.

It is also known that early integration of palliative care delivered in the ambulatory setting is associated with improved quality of life, decreased depression, and prolonged survival (Jackson et al., 2013). Helping patients to understand their illness is serious and to develop an understanding of their prognosis can be achieved by incorporating a step-wise, partnered approach using communication skills. Jackson and colleagues (2013) developed a series of steps that can be tailored and taught to health-care providers about how to communicate about serious illness and prognosis. These five steps for clinicians include preparation, assessment, inquiry, judging readiness, and delivering information:

Step 0: Prepare oneself with accurate information about a person’s likely life expectancy and the course of illness.

Step 1: Assess the patient’s prognostic awareness. Ask the question, "What is your sense of how you are doing?"

Step 2: Inquire whether the person can imagine a poorer health state. Ask the question, "What would it be like if you got sicker?"

Step 3: Judge patient readiness and clinical urgency. Consider, "Do I need to discuss the patient’s condition and prognosis now?"

Step 4: Deliver information about the patient’s condition (prognosis) tailored to patient readiness and clinical urgency.

Recently, Cardona-Morrell and Hillman (2015) published a clinical decision aid to assist in identifying clinical and demographic factors in hospitalized terminal patients who may benefit from a conversation about care goals. The authors developed an instrument—Criteria for Screening and Triaging to Appropriate ALternative Care (CriSTAL)—based on an extensive review of health-care professional literature. The authors plan to test a list of 29 possible predictors of death within 30 days to 12 weeks in a retrospective case-control medical record review of patients who have died of an emergency in an academic medical center. Examining this checklist in palliative care patients may be an appropriate use of these criteria.

Understanding patients’ and their families’ concerns in addition to their physical needs will enable earlier palliative care referrals and improved health outcomes, including quality of life. The instruments and educational tools described here are valuable and worth considering for use in practice. Such tools may promote earlier palliative care referral and encourage the use of more appropriate supportive care services. Other tools are available and have been documented and reviewed in the literature (Cardona-Morrell & Hillman, 2015); future research should evaluate the usefulness of these tools in active palliative care consultative practices.

Taking an individual’s preferences for palliative and end-of-life care into account is essential, to be sure that their wishes can be honored by health-care professionals.

## References

[1] Cardona-Morrell Magnolia, Hillman Ken (2015). Development of a tool for defining and identifying the dying patient in hospital: Criteria for Screening and Triaging to Appropriate aLternative care (CriSTAL).. *BMJ supportive & palliative care*.

[2] Fischer Stacy M, Gozansky Wendolyn S, Sauaia Angela, Min Sung-Joon, Kutner Jean S, Kramer Andrew (2006). A practical tool to identify patients who may benefit from a palliative approach: the CARING criteria.. *Journal of pain and symptom management*.

[3] Glare Paul, Virik Kiran, Jones Mark, Hudson Malcolm, Eychmuller Steffen, Simes John, Christakis Nicholas (2003). A systematic review of physicians' survival predictions in terminally ill cancer patients.. *BMJ (Clinical research ed.)*.

[4] Humphreys Jessi, Harman Stephanie (2014). Late referral to palliative care consultation service: length of stay and in-hospital mortality outcomes.. *The Journal of community and supportive oncology*.

[5] (2014). Dying in America: Improving Quality and Honoring Individual Preferences Near the End of Life.. *Institute of Medicine*.

[6] Jackson Vicki A, Jacobsen Juliet, Greer Joseph A, Pirl William F, Temel Jennifer S, Back Anthony L (2013). The cultivation of prognostic awareness through the provision of early palliative care in the ambulatory setting: a communication guide.. *Journal of palliative medicine*.

[7] (2013). Clinical Practice Guidelines for Quality Palliative Care.. *National Consensus Project*.

[8] (2014). Oncology Nursing Society Position Statement on Palliative Care for People With Cancer.. *Oncology Nursing Society*.

[9] Scarborough Bethann, Sean Morrison Senior Associate Editor R (2014). Integrating palliative and cancer care.. *Journal of palliative medicine*.

[10] Smith Thomas J, Temin Sarah, Alesi Erin R, Abernethy Amy P, Balboni Tracy A, Basch Ethan M, Ferrell Betty R, Loscalzo Matt, Meier Diane E, Paice Judith A, Peppercorn Jeffrey M, Somerfield Mark, Stovall Ellen, Von Roenn Jamie H (2012). American Society of Clinical Oncology provisional clinical opinion: the integration of palliative care into standard oncology care.. *Journal of clinical oncology : official journal of the American Society of Clinical Oncology*.

[11] Teno Joan M, Gozalo Pedro L, Bynum Julie P W, Leland Natalie E, Miller Susan C, Morden Nancy E, Scupp Thomas, Goodman David C, Mor Vincent (2013). Change in end-of-life care for Medicare beneficiaries: site of death, place of care, and health care transitions in 2000, 2005, and 2009.. *JAMA*.

[12] von Gunten Editor-In-Chief Charles F (2014). Prognosis: how long do we wait for the doctor?. *Journal of palliative medicine*.

[13] Weissman David E, Meier Diane E (2011). Identifying patients in need of a palliative care assessment in the hospital setting: a consensus report from the Center to Advance Palliative Care.. *Journal of palliative medicine*.

